# Acute Inducible Ablation of GRP78 Reveals Its Role in Hematopoietic Stem Cell Survival, Lymphogenesis and Regulation of Stress Signaling

**DOI:** 10.1371/journal.pone.0039047

**Published:** 2012-06-18

**Authors:** Shiuan Wey, Biquan Luo, Amy S. Lee

**Affiliations:** Department of Biochemistry and Molecular Biology, University of Southern California Keck School of Medicine, University of Southern California Norris Comprehensive Cancer Center, Los Angeles, California, United States of America; Baylor College of Medicine, United States of America

## Abstract

GRP78, a master regulator of the unfolded protein response (UPR) and cell signaling, is required for inner cell mass survival during early embryonic development. However, little is known about its role in adult hematopoietic stem cells (HSCs) and hematopoiesis. Here we generated a conditional knockout mouse model that acutely deletes *Grp78* in the adult hematopoietic system. Acute GRP78 ablation resulted in a significant reduction of HSCs, common lymphoid and myeloid progenitors, and lymphoid cell populations in the mutant mice. The GRP78-null induced reduction of the HSC pool could be attributed to increased apoptosis. Chimeric mice with *Grp78* deletion only in the hematopoietic cells also showed a loss of HSCs and lymphopenia, suggesting a cell intrinsic effect. Analysis of GRP78 deficient bone marrow (BM) cells showed constitutive activation of all the major UPR signaling pathways, including activation of eIF2α, ATF6, *xbp-1* splicing, as well as caspase activation. A multiplex cytokine assay further revealed alteration in select cytokine and chemokine serum levels in the mutant mice. Collectively, these studies demonstrate that GRP78 plays a pleiotropic role in BM cells and contributes to HSC survival and the maintenance of the lymphoid lineage.

## Introduction

Hematopoietic stem cells (HSCs) are multipotent stem cells that can differentiate to give rise to all lineages of mature blood cells and also self-renew. Self-renewal is a biological process where stem cells give rise to daughter cells that have the same potential as the original cell, including differentiation into the multiple lineages. In the adult hematopoietic system, regulation of HSC survival, self-renewal and differentiation is regulated both by intrinsic gene expression in a cell autonomous manner and also extrinsic cues from the microenvironment such as the stem cell niche [1], [2].

GRP78, also known as BiP/HSPA5, is an essential endoplasmic reticulum (ER) molecular chaperone protein and a master regulator of ER homeostasis [3]–[6]. The ER is the essential cellular organelle for proper folding and modification of secretory and membrane bound proteins. Metabolic, environmental and viral infection can result in ER stress. Upon ER stress, the unfolded protein response (UPR) is activated as an adaptive response to maintain cellular homeostasis [7]–[9]. UPR signaling is mediated by three sensor molecules, namely, PKR-like ER kinase (PERK), inositol-requiring enzyme 1 (IRE1α) and activating transcription factor 6 (ATF6), which are associated with GRP78 and retained inactive under normal, unstressed conditions. Upon activation, PERK phosphorylates eIF2α, which in turn inhibits general protein translation and activates C/EBP homologous protein (CHOP), which is a marker for ER stress-induced apoptosis. IRE1α is an endoribonuclease that upon activation initiates the splicing of the mRNA encoding X-box-binding protein 1 (XBP-1). Spliced XBP-1 is a potent transcriptional activator that upregulates the transcription of a subset of UPR related genes involved in protein folding, maturation and degradation in the ER. Activated ATF6 translocates from the ER to the Golgi, where it is cleaved by S1P/S2P proteases and generates an active transcription factor for induction of ER chaperone genes such as *Grp78* and other UPR targets. The role of the UPR has expanded beyond folding proteins in ER and is an important factor in regulating cell death and survival [8]–[10].

To study the *in vivo* function of GRP78, mouse models were constructed with heterozygous and homozygous knockout of the *Grp78* allele [11]. The heterozygous mice expressing 50% of the wild-type (WT) GRP78 were phenotypically normal, and showed no spontaneous activation of the UPR in embryos and fibroblasts derived from these mice. In contrast, *Grp78^−/−^* embryos demonstrated pre-implantation lethality. The GRP78 null embryos showed a dramatic reduction in proliferation, and strikingly, a massive increase in apoptosis in the inner cell mass, which is the precursor of embryonic stem cells [11]. This suggested that GRP78 may be important for stem cell survival.

GRP78 is expressed in primitive hematopoietic cells in leukemic disorders and required for leukemogenesis [12], [13]. However, little is known about the role of GRP78 in normal HSC and hematopoietic homeostasis. Here we report the generation of an inducible knockout mouse model by breeding *Grp78^f/f^* mice [11], [14] with *Mx-1-Cre* transgenic mice [15] to produce *Grp78^f/f^;Mx-1-Cre* (*c78^f/f^*) mice which allow acute, inducible and efficient deletion of GRP78 in the hematopoietic system through polyinosinic-polycytidylic acid (pI.pC) administration. Here we report that acute GRP78 deficiency in the hematopoietic system resulted in enhanced apoptotic death of the primitive HSC-enriched Lin^-^c-Kit^+^Sca-1^+^ (LSK) cell population and altered hematopoiesis with pronounced lymphopenia. The loss of HSCs and lymphopenia were also observed in chimeric mice that were deficient of GRP78 only in the hematopoietic cells, suggesting a cell autonomous effect. Furthermore, GRP78-null bone marrow (BM) cells showed constitutive activation of all three branches (ATF6, IRE1α and PERK) of UPR signaling suggestive of ER stress upon acute GRP78 ablation. Additionally, the serum levels of specific cytokines and chemokines were altered in the *c78^f/f^* mice. Our studies provide direct evidence that GRP78 is required to maintain the UPR signaling cascade in an inactive form in the hematopoietic system *in vivo* and that GRP78 contributes to HSC survival, lymphogenesis and hematopoietic homeostasis.

## Results

### Creation of a Mouse Model with Conditional Deletion of *Grp78* in the Hematopoietic System

**Figure 1:**
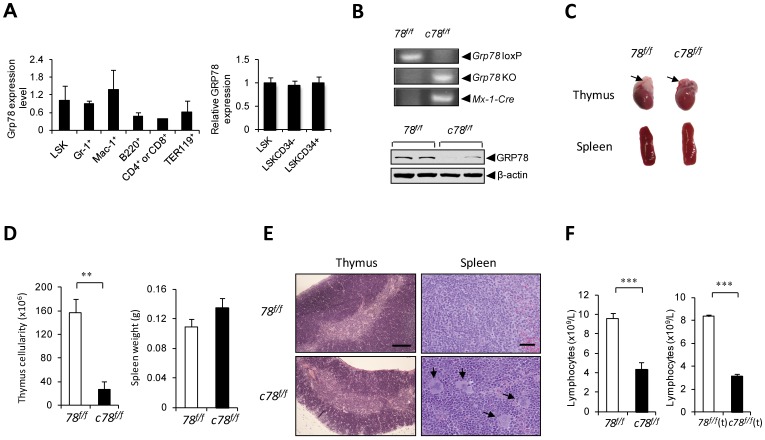
GRP78 deficiency leads to lymphopenia. A: GRP78 expression in WT BM subpopulations. (Left) *Grp78* mRNA expression in WT BM subpopulations measured by quantitative real-time PCR. The experiments were performed in duplicates; each replicate contains pooled BM from two WT mice. (Right) GRP78 expression in LSKCD34^−^ and LSKCD34^+^ subpopulations in WT mice (n = 4) measured by flow cytometry. The bar graph represents the medium intensities of GRP78 staining with LSK cells set as 1. B: (Upper) Representative PCR genotyping results from *78^f/f^* and *c78^f/f^* BM 6 days post completion of pI.pC treatment. (Lower) Western blot results for detection of GRP78 protein level in the BM performed in duplicates. C: Organ size and morphology from mice of the indicated genotypes. Arrows on top of the heart indicate thymus. D: Quantitation of the thymus cellularity (n = 4 for *78^f/f^*, n = 4 for *c78^f/f^*) and spleen weight (n = 14 for *78^f/f^*, n = 20 for *c78^f/f^*). E: H&E staining of paraffin sections of thymus and spleen of *78^f/f^* and *c78^f/f^* mice. Arrows indicate megakaryocytes in the spleen. The scale bar represents 200 µm in thymus and 20 µm in spleen. F: Peripheral lymphocyte count using complete blood count analysis with tail peripheral blood from (Left) *78^f/f^* (n = 12), *c78^f/f^* (n = 16) mice and (Right) *78^f/f^*(t) (n = 3), *c78^f/f^*(t) (n = 3) chimeric mice. All data are presented as mean ± s.e (**P<0.01, ***P<0.001, Student’s *t* test).

To identify the expression pattern of GRP78 in WT mouse BM cells, we performed real-time quantitative PCR and flow cytometric analyses with subpopulations of BM cells. As an essential ER chaperone, GRP78 was expressed in all six tested subpopulations including LT-HSC enriched Lin^-^c-Kit^+^Sca-1^+^CD34^−^ (LSKCD34^−^) and ST-HSC enriched LSKCD34^+^ cells (Figure 1A). To examine the role of GRP78 in the hematopoietic system, *Grp78^f/f^* mice were crossed with a pI.pC inducible *Mx-1-Cre* transgenic mouse line [15] that allows *Grp78* to be acutely deleted in the hematopoietic system. In this study, *Grp78* was deleted in the hematopoietic system of 6–8 week old adult *Grp78^f/f^;Mx-1-Cre* mice (referred to as *c78^f/f^*) upon administration of pI.pC every other day for a total of 7 injections to activate *Cre* expression. Littermates without the *Cre* transgene (*Grp78^f/f^*), which are phenotypically equivalent to animals with a WT *Grp78* allele, served as WT normal controls and were also injected with pI.pC. The status of *Grp78* deletion in BM cells was validated by PCR (Figure 1B). Western blot analysis of BM cells confirmed that GRP78 expression was nearly completely ablated in the *c78^f/f^* mice (Figure 1B). All analysis was carried out 6 days post 7 injections of pI.pC, due to onset of lethality of *c78^f/f^* mice at later times (data not shown).

### Depletion of GRP78 in the Hematopoietic System Leads to Intrinsic Alteration of Hematopoiesis

Upon the Mx-1-Cre mediated acute depletion of GRP78 in the *c78^f/f^* mice, we observed a substantially smaller thymus corresponding to an 80% decrease in thymus cellularity (P<0.01), while the spleen size was comparable to *78^f/f^* siblings (Figure 1C and D). H&E staining of the paraffin tissue sections showed hypocellularity in the thymus, and signs of extramedullary hematopoiesis in the spleen as evidenced by the presence of megakaryocytes (Figure 1E). Furthermore, complete blood count analysis from tail peripheral blood demonstrated a 60% decrease in lymphocyte number (P<0.001) in the *c78^f/f^* mice compared to *78^f/f^* (Figure 1F). Since Mx-1-Cre mediated *Grp78* deletion occurs in the whole BM cells and the microenvironment, to address whether the effects observed in the *c78^f/f^* mice is cell intrinsic, we isolated total BM cells of *c78^f/f^* mice and transplanted them into lethally irradiated *78^f/f^* siblings. As control, BM cells of *78^f/f^* mice were also transplanted into another set of lethally irradiated *78^f/f^* littermates. These mice were referred to as *c78^f/f^*(t) and *78^f/f^*(t) respectively below. Two months following transplantation to allow full BM reconstitution, both sets of recipient mice were intraperitoneally injected with pI.pC to induce Mx-1-Cre mediated deletion, and were analyzed 6 days after 7 injections. Thus, in the *c78^f/f^*(t) mice, GRP78 would only be depleted in the hematopoietic cells. Pronounced peripheral blood lymphopenia was also observed in the *c78^f/f^*(t) mice (Figure 1F), indicating that the lymphopenia is cell autonomous.

**Figure 2:**
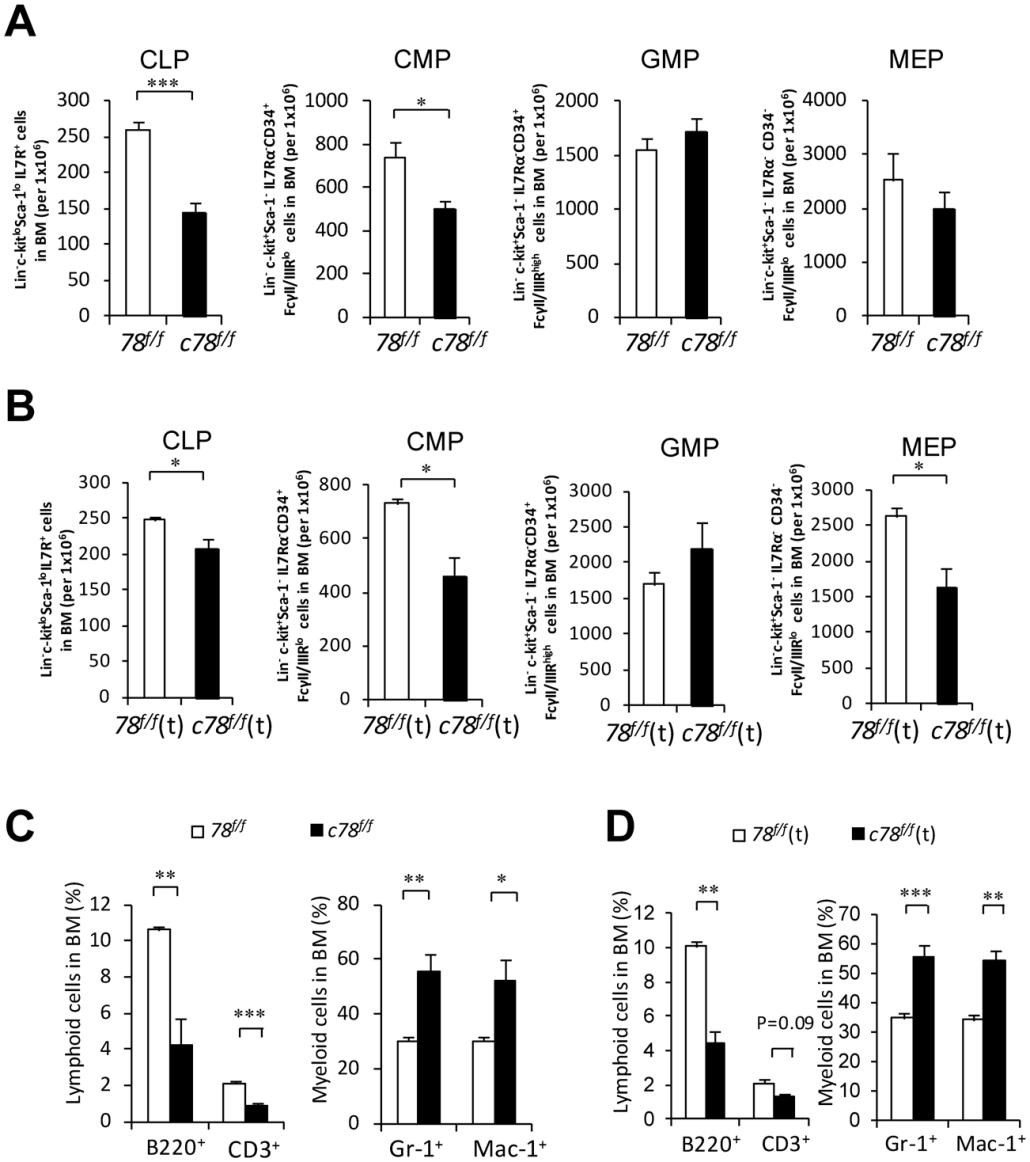
Deletion of GRP78 in the hematopoietic system leads to altered hematopoiesis. A: Quantitation of flow cytometric analysis of lymphoid and myeloid progenitors including common lymphoid progenitor (CLP), common myeloid progenitor (CMP), granulocyte-monocyte progenitor (GMP) and megakaryocyte-erythroid progenitor (MEP) from *78^f/f^* (n = 6) and *c78^f/f^* (n = 6) mice. B: Quantitation of flow cytometric analysis of lymphoid and myeloid progenitors including common lymphoid progenitor (CLP), common myeloid progenitor (CMP), granulocyte-monocyte progenitor (GMP) and megakaryocyte-erythroid progenitor (MEP) from *78^f/f^*(t) (n = 3) and *c78^f/f^*(t) (n = 3) mice. C: Quantitation of lymphoid and myeloid cells from flow cytometric analysis with BM cells in *78^f/f^* and *c78^f/f^* mice using lineage markers B220, CD3, Gr-1 and Mac-1 (n = 4 for each genotype). D: Quantitation of lymphoid and myeloid cells from flow cytometric analysis with BM cells in *78^f/f^*(t) and *c78^f/f^*(t) mice using lineage markers B220, CD3, Gr-1 and Mac-1 (n = 3 for each genotype). All data are presented as mean ± s.e (*P<0.05, **P<0.01, ***P<0.001, Student’s *t* test).

To explain the lymphopenia in the peripheral blood, the myeloid and lymphoid progenitor cell populations in the BM were examined. In the *c78^f/f^* mice, a significant 45% decrease in both the common lymphoid progenitor (CLP) enriched Lin^-^c-Kit^lo^Sca-1^lo^IL-7Rα^+^ cells and 33% decrease in the common myeloid progenitor (CMP) enriched Lin^-^c-Kit^+^Sca-1^-^IL-7Rα^-^CD34^+^FcγII/IIIR^lo^ cells were observed, whereas there was a modest (21%) decrease which had not reached statistical significance in the megakaryocyte-erythroid progenitor (MEP) enriched Lin^-^c-Kit^+^Sca-1^−^IL-7Rα^-^CD34^−^FcγII/IIIR^lo^ cells (Figure 2A). In contrast, the granulocyte-monocyte progenitor (GMP) enriched Lin^-^c-Kit^+^Sca-1^−^IL-7Rα^-^CD34^+^FcγII/IIIR^high^ cell population was comparable between the *c78^f/f^* mice and their *78^f/f^* littermates. Cytometric analysis confirmed similar reduction of GRP78 expression level in all four progenitor populations (Figure S1).

In agreement with the results in *c78^f/f^* mice, we also observed a significant decrease in CLP enriched Lin^-^c-Kit^lo^Sca-1^lo^IL-7Rα^+^ cells in the *c78^f/f^*(t) mice, however, to a lesser extent (Figure 2B). Consistent to what was observed in *c78^f/f^* mice, a significant decrease in CMP enriched Lin^-^c-Kit^+^Sca-1^−^IL-7Rα^-^CD34^+^FcγII/IIIR^lo^ cells was also found in *c78^f/f^*(t) mice. In addition, the MEP enriched Lin^-^c-Kit^+^Sca-1^−^IL-7Rα^-^CD34^−^FcγII/IIIR^lo^ cells were also significantly reduced in the *c78^f/f^*(t) mice whereas in the *c78^f/f^* mice, the MEP population was only modestly reduced. The GMP enriched Lin^-^c-Kit^+^Sca-1^−^IL-7Rα^-^CD34^+^FcγII/IIIR^high^ cell population in the *c78^f/f^*(t) mice was comparable to the *78^f/f^*(t) mice (Figure 2B) which was similar to the results in *c78^f/f^* mice.

We further analyzed the mature cells in the BM of the *c78^f/f^* and *78^f/f^* mice. A 60% drop in lymphoid cells and a 2-fold increase of myeloid cells was observed in *c78^f/f^* mice compared to *78^f/f^* littermates, as evidenced by a decrease in both the B220^+^ and CD3^+^ cells and an increase in Gr-1^+^ and Mac-1^+^ cells (Figure 2C). Consistent with *c78^f/f^* results, we observed a 60% decrease in B220^+^ lymphoid cells (P<0.01) and a 1.6-fold increase in Gr-1^+^ and Mac-1^+^ myeloid cells (P<0.01) in the *c78^f/f^*(t) mice (Figure 2D). Collectively, these observations suggest that GRP78 deficiency in the hematopoietic system leads to a reduction of the lymphoid lineage from progenitor to mature cells, at least in part in a cell intrinsic manner.

### Acute GRP78 Ablation Decreases Primitive Cell Pool Through Increased Apoptosis

**Figure 3:**
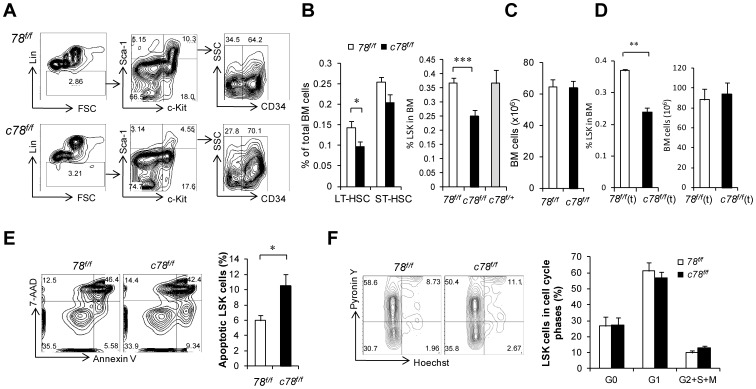
GRP78 deficiency in the BM reduced HSC-enriched population through increased cell death. A: Representative flow cytometric analysis with BM cells using Lin, c-Kit, Sca-1 and CD34. B: ?Left) Quantitation of flow cytometric analysis of Lin^-^c-Kit^+^Sca-1^+^CD34^−^ (LT-HSC) and Lin^-^c-Kit^+^Sca-1^+^CD34^+^ (ST-HSC) populations in the BM (n = 6 for *78^f/f^*, n = 6 for *c78^f/f^*). (Right) Quantitation of flow cytometric analysis of HSC-enriched LSK population in the BM (n = 12 for *78^f/f^*, n = 16 for *c78^f/f^*, n = 4 for *c78^f/+^*). C: Total BM cell number from *78^f/f^* (n = 11) and *c78^f/f^* (n = 16) mice. D: Quantitation of LSK percentage and total BM cell number from *78^f/f^*(t) and *c78^f/f^*(t) mice (n = 3 for each analysis). E: (Left) Representative flow cytometric analysis of apoptotic LSK cells using Annexin V and 7-AAD. (Right) Summary of apoptotic LSK cells (Annexin V^+^7-AAD^–^) (n = 5 for *78^f/f^*, n = 6 for *c78^f/f^*). F: (Left) Representative flow cytometric analysis of LSK cell cycle status by Hoechst and Pyronin Y staining. (Right) Summary of cell cycle distribution of LSK cells from *78^f/f^* (n = 4) and *c78^f/f^* (n = 4) mice. All data are presented as mean ± s.e (*P<0.05, **P<0.01, Student’s *t* test).

Upon acute depletion of GRP78 in adult primitive hematopoietic cells, we observed a reduction in both the LT-HSC enriched Lin^−^c-Kit^+^Sca-1^+^CD34^−^ (LSKCD34^−^) and ST-HSC enriched LSKCD34^+^ cells (Figure 3A and B) in the BM. Corresponding with this was a 25% reduction in LSK cell population in the BM (P<0.01) (Figure 3A and B). Furthermore, the LSK population in the heterozygous *Grp78* knockout (*c78^f/+^*) mice did not show a reduction of LSK population (Figure 3B) suggesting the LSK reduction in the *c78^f/f^* mice was not due to the presence of the *Cre* allele. In addition, there was no difference in the total BM cell number between *78^f/f^* and *c78^f/f^* mice (Figure 3C). This suggests that the total number of primitive hematopoietic cells in *c78^f/f^* mice BM is lower compared to *78^f/f^* controls. The reduction of primitive hematopoietic cells was also observed in the *c78^f/f^*(t) mice. While the total BM cellularity was the same, we observed a 35% reduction of HSC-enriched LSK (P<0.01) in the BM of the *c78^f/f^*(t) mice upon pI.pC injection (Figure 3D). The magnitude of LSK reduction was in the same range as the *c78^f/f^* mice, suggesting that the loss of LSK cells upon GRP78 depletion is cell intrinsic.

To determine whether the decrease of the primitive hematopoietic cells in the BM of the *c78^f/f^* mice was due to increased cell death or decreased proliferation, the apoptotic and cell cycle profiles in LSK cells were analyzed. Apoptotic LSK cells were examined by flow cytometric analysis of Annexin V and 7-AAD staining. Our results showed that the apoptotic LSK cells increased from 6% in *78^f/f^* mice to 10.5% in the *c78^f/f^* mice (P<0.05) (Figure 3E), while cell cycle distribution analyzed by Hoechst/Pyronin Y staining showed no significant difference between *c78^f/f^* and *78^f/f^* mice (Figure 3F). This suggests that GRP78 contributes to the maintenance and survival of adult HSCs as acute GRP78 ablation results in a decrease in the primitive hematopoietic cells at least in part through enhanced apoptotic cell loss.

### Acute Ablation of GRP78 in BM Cells Leads to Constitutive UPR Activation

**Figure 4:**
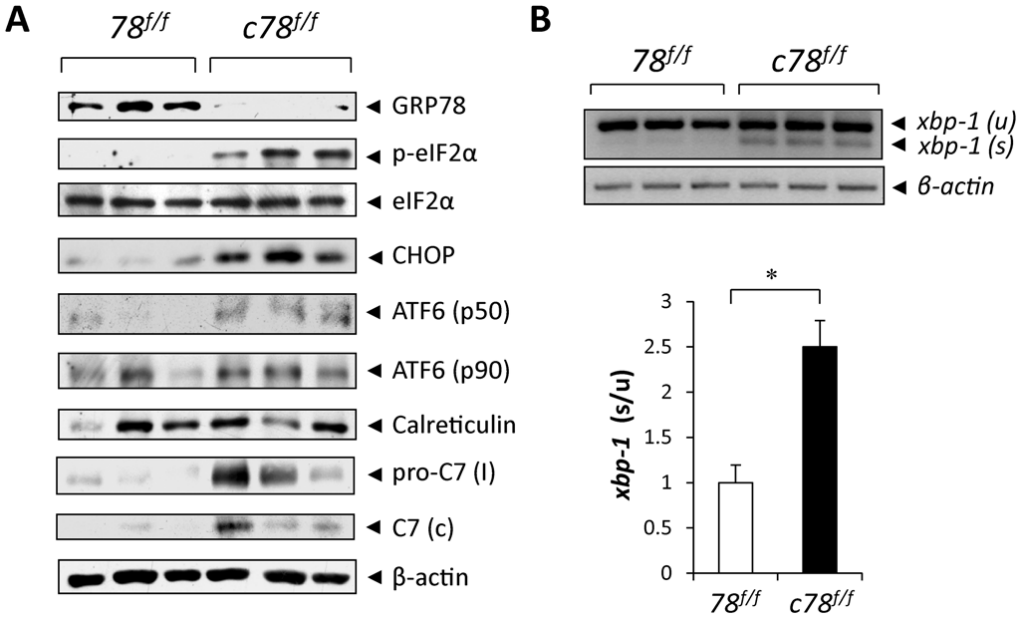
Knockout of GRP78 in BM cells activates UPR signaling pathways. A: Western blot results using BM cell lysates (n = 3 for each genotype) for detection of GRP78, phospho-eIF2α, total eIF2α, CHOP, ATF6 (p50), ATF6 (p90), calreticulin, pro-caspase-7 intermediate, cleaved caspase-7 and β-actin. B: (Upper panel) RT-PCR results for detection of *xbp-1* spliced [*xbp-1(s)*], *xbp-1* unspliced [*xbp-1(u)*] and *β-actin* mRNA levels from BM cells of *78^f/f^* and *c78^f/f^* mice (n = 3 for each genotype). The PCR image was inverted for better clarity. (Lower panel) Quantitation of the ratio of *xbp-1(s)* to *xbp-1(u)*. The average ratio of *xbp-1(s)*/*xbp-1(u)* in *78^f/f^* was set as 1. The data is presented as mean ± s.e. (*P<0.05, Student’s *t* test).

GRP78 is a master regulator of the UPR such that the transmembrane ER stress signaling sensors (PERK, IRE and ATF6) are maintained in an inactive form by GRP78 in non-stressed cells [7]–[9]. Upon onset of the UPR, GRP78 is titrated away, and depending on the severity of the stress stimuli, pro-survival as well as apoptotic components of the UPR will be activated. To investigate the role of GRP78 in maintaining hematopoietic homeostasis, cell lysates were prepared from the BM cells of *78^f/f^* and *c78^f/f^* mice and subjected to Western blot analysis, which confirmed efficient deletion of *Grp78* as the GRP78 level was greatly depleted in *c78^f/f^* BM cells (Figure 4A). One of the indicators of activation of the PERK branch of UPR signaling is phosphorylation of eIF2α, which was at high levels in the *c78^f/f^* compared to *78^f/f^* controls while the total eIF2α level remained the same (Figure 4A). CHOP, which is a downstream target of p-eIF2α signaling, was greatly induced in the BM cells of *c78^f/f^* mice. Additionally, activation of the ATF6 branch was observed in *c78^f/f^* mice as evidenced by the enhanced presence of the activated, nuclear form of ATF6 (p50). For examining activation of the IRE-1 pathway, we measured the mRNA splicing of the XBP-1 transcription factor which is a substrate of activated IRE-1. There was a 2.5-fold increase of spliced over unspliced *xbp-1* mRNA level in the BM cells of *c78^f/f^* mice compared to *78^f/f^* littermates (Figure 4B). The level of another ER chaperone, calreticulin, as well as β-actin remained the same (Figure 4A).

Caspase-7 has been shown to be associated with the ER and GRP78 maintains it in an inactive form in non-stressed cells [16]. Upon activation, caspase-7 is converted to an intermediate precursor form [pro-C7 (I)], followed by cleavage generating the active form of caspase-7 [C7(c)]. In the BM cells of *c78^f/f^* mice, we observed varying levels of increase for the intermediate pro-caspase-7 and cleaved caspase-7 (Figure 4A). Thus, depletion of GRP78 in the BM cells of cKO mice triggers ER stress as evidenced by the constitutive activation of all three branches (PERK, ATF6 and IRE-1) of the UPR signaling cascade. Some cell types are able to withstand ER stress as the UPR confers protective measures against the loss of GRP78, however, for some other cell types, such as the LSKs, the induction of the pro-apoptotic branch of the UPR may lead to apoptosis in the *c78^f/f^* mice.

### GRP78 Deficient Mice Exhibit Differential Expression of Cytokines and Chemokines

**Figure 5:**
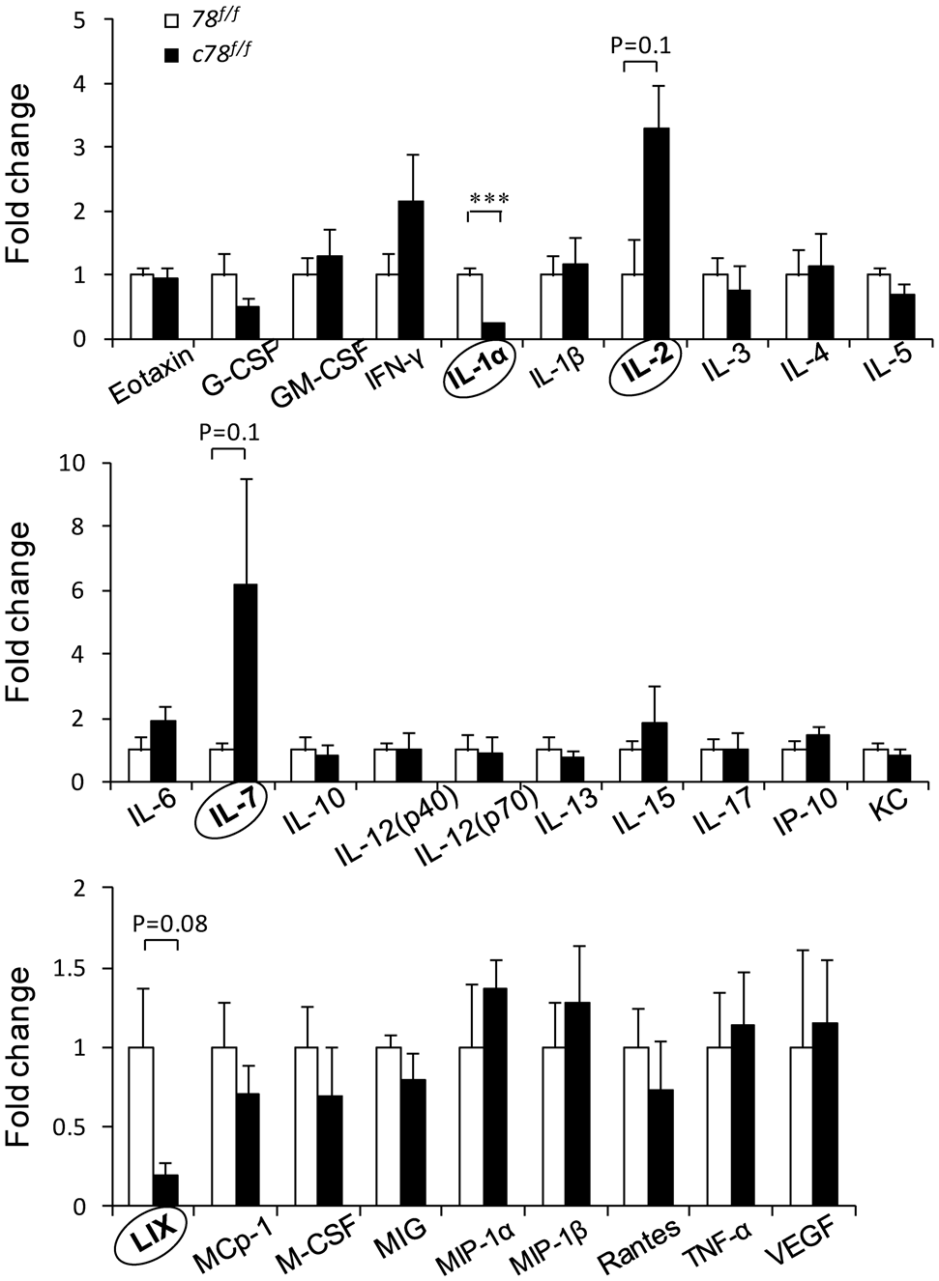
Differential cytokine and chemokine expression in serum of GRP78 deficient mice. (Upper, middle, lower) bar graphs demonstrating the differential expression of cytokines and chemokines in the serum of *78^f/f^* (n = 3) and *c78^f/f^* (n = 3) mice. The ones that exhibit major difference between the *c78^f/f^* and *78^f/f^* mice are circled and bold highlighted. The data is presented as mean ± s.e. The P values are indicated (***P<0.001, Student’s *t* test).

Cytokines and chemokines are important factors for maintaining normal hematopoiesis. The creation of the *c78^f/f^* mouse model provides an opportunity to investigate the effect of acute GRP78 depletion on various cytokine and chemokine production. Analysis of the differential expression of cytokines and chemokines in the serum of *78^f/f^* and *c78^f/f^* mice revealed several interesting candidate molecules that may provide further explanation for the altered hematopoiesis observed in the GRP78-null mice. Out of the 29 cytokines/chemokines examined, the most differentially expressed cytokine between *78^f/f^* and *c78^f/f^* mice was interleukin-1 alpha (IL-1α), where the IL-1α level in *c78^f/f^* mice was 4-fold lower than in *78^f/f^* siblings (P<0.0001) (Figure 5). IL-1α is a multifunctional proinflammatory cytokine that can affect many cell types [17]. It promotes lymphocyte proliferation *in vivo* and has been reported to have synergistic interactions with TNF-α for its requirement in the expression of CD25 (IL-2α receptor chain) and maturation of thymocytes [18]. The chemokine lipopolysaccharide-inducible CXC chemokine (LIX) also exhibited a 5-fold decrease in the *c78^f/f^* mice compared to *78^f/f^* siblings. LIX is usually associated with cell migration and activation in neutrophils [19] but is also found to have a role in hematopoietic stem cell maintenance [20]. The reduction of LIX levels and corresponding decrease in hematopoietic stem cell populations in *c78^f/f^* mice suggests the potential that the regulation of GRP78-null induced HSC reduction may be in part through LIX chemokine interaction.

Interestingly, there was a 6-fold increase in interleukin-7 (IL-7) serum levels in the *c78^f/f^* mice compared to the *78^f/f^* mice. IL-7 is produced by BM and thymic stromal cells and is the major lymphopoietic and thymopoietic cytokine. IL-7 has been identified as a non-redundant cytokine essential for proper T and B cell development [21], [22], induces proliferation and differentiation of immature thymocytes [23], and protects thymocytes from apoptosis by induction of Bcl-2 expression [24]. In the *c78^f/f^* mice, we observed a loss of lymphocytes in the peripheral blood and BM, and a severe loss of thymocytes indicating a significantly compromised lymphopoiesis. Thus, the increase in IL-7 level may be part of a homeostatic compensatory response to T lymphocyte depletion in the *c78^f/f^* mice. Likewise, the level of interleukin-2 (IL-2), the cytokine immune system signaling molecule important for the proliferation and maturation of responsive lymphocytes [25], was about three times higher in the *c78^f/f^* mice (Figure 5). Thus, the alterations appear to be mainly in lymphocyte specific factors affecting lymphogenesis.

## Discussion

While GRP78 is well-established to exhibit anti-apoptotic properties and plays a crucial role in early embryogenesis, solid tumor progression, oncogenesis, neurodegeneration, and atherosclerosis [9], [26]–[28], the function of GRP78 in the hematopoietic system is just emerging. Recent reports show that GRP78 expression is upregulated in various forms of human leukemia and implicated as a causative factor for therapeutic resistance and early relapse [13], [29], [30]. Through creation of a biallelic conditional knockout mouse of GRP78 and PTEN in the hematopoietic system, it was discovered that while heterozygous knockdown of GRP78 in the BM does not result in any detectable hematopoietic abnormality, GRP78 haploinsufficiency in the PTEN null mice is sufficient to restore the HSC population and suppress leukemic blast expansion at least in part through suppression of PI3K/AKT signaling [13]. Despite these new advances, the role of GRP78 in normal hematopoiesis remains to be determined.

**Figure 6:**
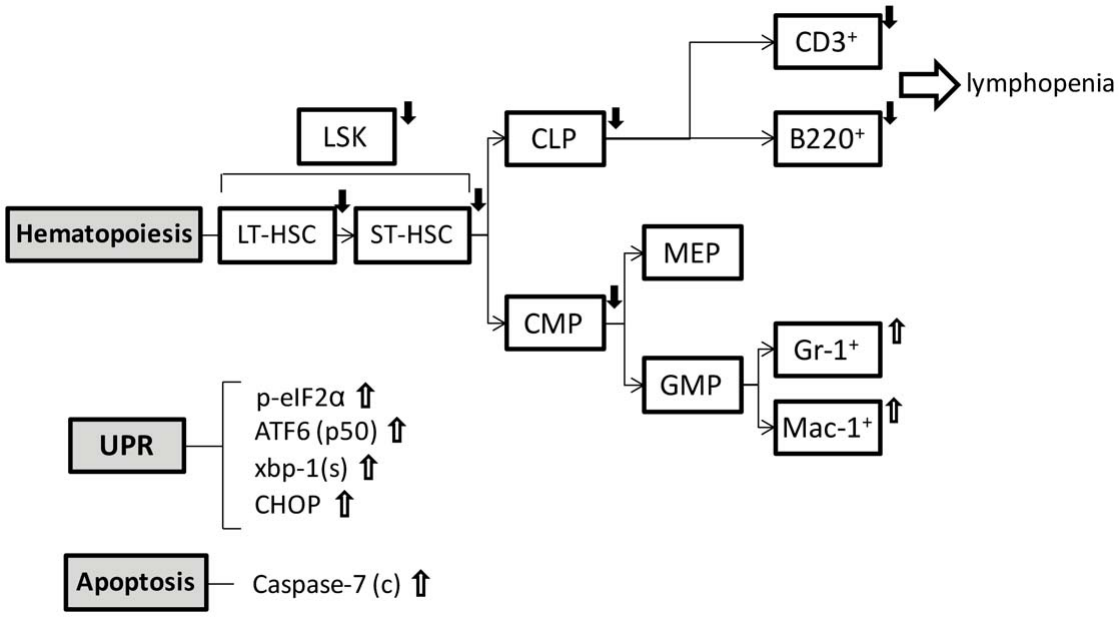
Summary diagram of alteration of hematopoiesis, UPR signaling and apoptosis in *Grp78* conditional knockout in the hematopoietic system. GRP78 depletion in the hematopoietic system leads to altered hematopoiesis, activated UPR signaling and enhanced apoptosis. Open arrows represent an increased level and closed arrows represent a decreased level.

Our investigation into the role of GRP78 in HSC stem cell survival, differentiation and hematopoiesis using a model of acute, near complete ablation of GRP78 in the BM, revealed several novel observations. In this study, we provide the first evidence that GRP78 depletion results in reduction of HSC population due to enhanced apoptosis in the primitive cell population, indicating GRP78 contributes to HSC survival and maintenance. Furthermore, Mx-1-Cre induced *Grp78* knockout resulted in reduction of lymphoid progenitor cells, corresponding to a decrease in lymphocytes both in BM and in circulation, and a severe reduction of thymocytes. In contrast, while a decrease in myeloid progenitor cells was observed in the *c78^f/f^* mice, there was an expansion of myeloid cells in the BM, which could be a compensatory response of the impaired lymphogenesis. A summary of the alterations in the hematopoietic lineages and signaling pathways of the *c78^f/f^* mice is shown in Figure 6. Since the phenotypes of the *c78^f/f^* mice were also observed in the chimeric mice where GRP78 was depleted only in the hematopoietic cells, this implies that alteration of hematopoiesis resulting from GRP78 deficiency was mediated at least in part in a cell intrinsic manner. In agreement that GRP78 is critical for the maintenance of cellular homeostasis, we observed constitutive activation of the three major branches of the UPR signaling in the BM cells in the *c78^f/f^* mice, as well as varying levels of caspase activation.

How does GRP78 depletion result in altered hematopoiesis? As an essential molecular chaperone and cell signaling regulator, GRP78 has the potential to impact hematopoiesis at multiple levels. As observed in early embryogenesis, GRP78 is required for the proliferation and survival of the inner cell mass [11]. Here, we also observed that GRP78 is required to maintain viability of the primitive HSCs. One possible explanation is the ability of GRP78 to exert multiple anti-apoptotic properties [9], [16]. Interestingly, while we detected constitutive activated signaling in all three branches, the PERK, ATF6 and IRE1α pathways, including induction of pro-apoptotic CHOP and some levels of caspase activation in the *c78^f/f^* BM cells, the total number of BM cells in the *78^f/f^* and *c78^f/f^* mice remained the same, suggesting that most of the BM cells were protected against the ER stress by the UPR or some other mechanisms. On the other hand, the enhanced apoptosis in the GRP78-null HSC-enriched population suggests that these cells may be particularly vulnerable to ER stress which could switch the cytoprotective functions of the UPR into apoptotic programs dependent on both extrinsic and intrinsic factors [7]–[9]. Secondly, GRP78 is an important factor for secretion efficiency of selective proteins [31]. As such, GRP78 regulates processing of antigens [32] and cytokine production and secretion [33]. Thirdly, in specific cell types, a subfraction of GRP78 can be localized to the cell surface acting as a co-receptor for cell signaling, impacting both proliferation and survival [34]. Interestingly, it is recently reported that cell surface GRP78, in complex with Cripto, maintains a small subset of LSK cells (cell surface GRP78^+^CD34^−^ LSK) in the hypoxic endosteal niche and prevents them from moving to the central marrow [35].

In our study, we observed decreased lymphoid cells in the peripheral blood and BM in the *c78^f/f^* mice. The decrease in lymphoid cells corresponds to the reduction of upstream CLP enriched population in the BM and a severe loss of thymus cellularity in *c78^f/f^* mice. Intriguingly, we observed a general increase in myeloid cells in the BM, whereas we detected a decrease in their CMP enriched population and no significant difference in the GMP enriched population. This indicates that the expansion of myeloid cells is not a consequence of increased upstream progenitor populations, but is mediated by other mechanisms, such as a compensatory effect of the reduction in lymphoid lineage, which remains to be resolved.

In addition to its well-known function as the immunoglobulin heavy chain binding protein, GRP78 has been reported to have immunomodulatory properties [33]. The multiplex cytokine assay showed that acute *Grp78* ablation led to a substantial decrease in IL-1α and LIX levels, while showing an increase in IL-2 and IL-6 levels. While the mechanism for the alterations remains to be determined, IL-1α is synthesized as a 33 kD molecule, which is proteolytically processed to the bioactive 17 kD form by calpain in a Ca^2+^ dependent manner [36]. Depletion of GRP78, an ER Ca^2+^ binding protein, in the *c78^f/f^* mice may alter the Ca^2+^ balance and disrupt the Ca^2+^ dependent cleavage of IL-1α. The decrease of LIX implicated in HSC cell maintenance raises the intriguing possibility of whether its downregulation in the *c78^f/f^* mice may contribute to the reduction of the HSC population and maintenance [20]. On the other hand, the increase in IL-2 and IL-7 levels may be part of a homeostatic, compensatory response to T lymphocyte depletion in the *c78^f/f^* mice. In agreement, HIV-1-mediated T cell depletion exhibited increased IL-7 production [37] and lymphopenic patients had higher levels of serum IL-7 [38]. As cytokines/chemokines can be produced by both hematopoietic and stromal cells, future studies are necessary to discern the modulatory effects of GRP78 depletion in the microenvironment.

As we report here that acute GRP78 ablation in the hematopoietic system results in reduction of HSC in BM, depletion of another ER chaperone, GRP94, significantly increases the HSC population [39]. Acute GRP94 elimination in the hematopoietic system led to an expansion of HSCs due to a loss of quiescence resulting from impaired interaction of HSCs and the BM niche [39]. Therefore, despite their shared function as ER chaperones, GRP78 and GRP94 play differential roles in the hematopoietic system. Our studies provide the first evidence that GPR78 contributes to HSC survival, regulates hematopoiesis and is key to maintaining ER stress signaling in an inactive form, suggesting that GRP78 plays a pleiotropic and important role in the hematopoietic system, which warrants further investigation. Another interesting point raised in these studies is the onset of lethality in some of the *c78^f/f^* mice within a few weeks after Mx-1-Cre-induction, accompanied by reduction in food intake, body weight loss and blood glucose (data not shown). We speculate that while *Grp78* inactivation caused by pI.pC inducible Mx-1-Cre is highly effective in the hematopoietic system, heterogeneous induced *Grp78* deletion in other organs may also come into play [15], leading to lethality. Future studies with systematic organ specific deletion of *Grp78* will address its precise role in organ maintenance and survival.

## Materials and Methods

### Ethics Statement

All protocols for animal use were reviewed and approved by the USC Institutional Animal Care and Use Committee. The animal assurance number is A3518-01. The protocol number is 9964.

### Mice

*Grp78^f/f^* mice in a mixed C57BL/6;129/Sv background [11], [14] were crossed with the transgenic *Mx-1-Cre* mice in a C57BL/6 background (Jackson Laboratory) to generate *Grp78^f/f^;Mx-1-Cre* mice. The schematic diagram of the breeding and maintenance strategy is depicted in Figure S2. Littermates that did not carry the *Cre* transgene (*78^f/f^*) were used as WT controls. Genotyping was performed as previously described [14]. To induce the genomic deletion, 6–8 week old mice were injected intraperitoneally with pI.pC (25 µg/g mouse body weight) every other day for 14 days. Mice were analyzed 6 days post 7 injections of pI.pC.

### RT-PCR and Real-time Quantitative PCR

Total RNA from BM samples were extracted and reverse-transcription was performed as described [40]. The primers used for RT-PCR for *xbp-1* are 5′-GAACCAGGAGTTAAGAACACG-3′ and 5′-AGGCAACAGTGTCAGAGTCC-3′ and those for β-actin are 5′-GACGGCCAGGTCATCACTAT-3′ and 5′-GTACTTGCGCTCAGGAGGAG-3′. To detect *Grp78* expression, real-time quantitative RT-PCR was performed. RNA was extracted from sorted populations of mouse whole BM cells and reverse-transcribed. cDNA samples were analyzed in triplicate with the SYBR Green Supermix (Quanta Biosciences, Gaithersburg, MD) according to manufacturer’s instructions. The primers used for mouse *Grp78* are 5′-TCTCCACGGCTTCCGATAAT-3′ and 5′-GTACCTTTGTCTTCAGCTGTCACTC-3′ and those used for *18 S RNA* are 5′-ACGGCCGGTACAGTGAAAC-3′ and 5′-GAGGGAGCTCACCGGG-3′.

### Multiplex Cytokine Assay of Serum

Six days post completion of the pI.pC administration interval, blood was collected from retro-orbital bleeding in Microtainer (Becton Dickinson) serum gel collection tubes and allowed to clot at room temperature for 30 mins and centrifuged at 4°C for 5 mins at 13,000 g. Serum was collected from the top of the tube followed by analysis as described [41]. A 23-plex mouse cytokine assay (Bio-Rad Laboratories) was carried out following manufacturer’s instructions (see File S1) and the results analyzed using the Bio-plex Manager software (Bio-Rad Laboratories).

### BM Transplantation

*Grp78^f/f^* mice were lethally irradiated at 9.5 Gy and subjected to BM transplantation assays. Within 24 hrs, 1×10^6^ total BM cells isolated from the *Grp78^f/f^;Mx-1-Cre* mice were injected through tail vein into individual lethally irradiated recipient mice. Similarly, BM cells isolated from the *Grp78^f/f^* mice were injected into individual irradiated recipient mice. pI.pC injection was administered 8 weeks after transplantation into both sets of mice.

### Flow Cytometry

BM single cell suspension was obtained and subjected to flow cytometric analysis as previously described [39]. The following antibodies from BD Pharmingen were used: Lineage (Lin; which consists of B220 (RA3-6B2), TER119 (TER119), CD4 (RM4–5), CD8 (53–6.7), Gr-1 (RB6-8C5), and Mac-1 (WT.5)), c-Kit (2B8), Sca-1 (D7), CD34 (RAM34), IL7Rα (SB/199), FcγRII/III (2.4G2) and CD3 (1F4). For intracellular GRP78 staining, BM cells stained with primitive hematopoietic cell surface markers were fixed with 2% paraformaldehyde on ice for 20 mins, and then permeablized with DPBS with 0.01% saponin for 30 mins at RT. 5% goat serum in DPBS with 0.01% saponin was used for blocking. GRP78 was stained with anti-GRP78 antibody (10 µg/mL; H129 from Santa Cruz Biotechnology) for 2 hrs at RT and AF488-conjugated secondary antibody derived in goat (5 µg/mL; A11008 from Life Technologies) for 30 mins at RT and were analyzed by LSR II flow cytometer (Becton Dickinson).

### Fluorescence Activated Cell Sorting

Primitive hematopoietic stem and progenitor cells and different lineages of BM cells were purified using a FACS Aria flow cytometer (Becton Dickinson) based on established cell surface phenotypes.

### Cell Cycle Analysis, Apoptosis Assays and Complete Blood Count

Hoechst 33342/Pyronin Y staining for cell cycle analysis, Annexin V/7-AAD staining for apoptosis assays and complete blood count were performed as previously described [39].

### Western Blot Analysis

Whole cell lysates were prepared from single-cell suspensions of BM cells and immunoblotted as described [13]. The primary antibodies used were as follows: monoclonal mouse anti-GRP78 (1∶2000) and monoclonal mouse anti-caspase-7 (1∶2000) are from BD Pharmingen. Rabbit anti-p-eIF2α (Ser51, 1∶1000), rabbit anti-eIF2α (1∶1000) are from Cell Signaling. Mouse anti-ATF6 (1∶200) is from Imgenex. Rabbit anti-calreticulin (1∶2000) is from Stressgen. Mouse anti-CHOP (1∶1000) is from Santa Cruz Biotechnology. Mouse anti-β-actin (1∶5000) is from Sigma.

### Statistical Analyses

For bar graphs, the unpaired 2-tailed Student’s *t* test was used to compute P values, and the error bars reflect standard error (s.e.) (*, P<0.05, **, P<0.01, ***, P<0.001).

## Supporting Information

Figure S1 **GRP78 knockout efficiency in lymphoid and myeloid progenitors.** Bar graph represents the medium intensities of total GRP78 staining measured by flow cytometry in common lymphoid progenitor (CLP), common myeloid progenitor (CMP), granulocyte-monocyte progenitor (GMP) and megakaryocyte-erythroid progenitor (MEP) from *78^f/f^* (n = 2) and *c78^f/f^* (n = 2) mice.
(TIF)

Figure S2 **The breeding scheme for the Grp78 conditional knockout mice.** The generation of parental *Grp78^f/f^* was described previously [11], [14]. *Grp78^+/+^;Mx-1-Cre* was commercially purchased from the Jackson Laboratory. The genotypes indicated with the gray shade were used in this study. The genetic background of the parental mouse strain is indicated below within the square brackets. The numbers below the genotypes indicate the expected probability of the indicated genotype among the offspring.
(TIF)

File S1 **Materials and Methods.**
(DOC)
