# Supplementary figures and images for: Acute Inducible Ablation of GRP78 Reveals Its Role in Hematopoietic Stem Cell Survival, Lymphogenesis and Regulation of Stress Signaling
Source: PLoS One. 2012 Jun 18;7(6):e39047. doi: 10.1371/journal.pone.0039047 (PMC3377598; doi:10.1371/journal.pone.0039047)

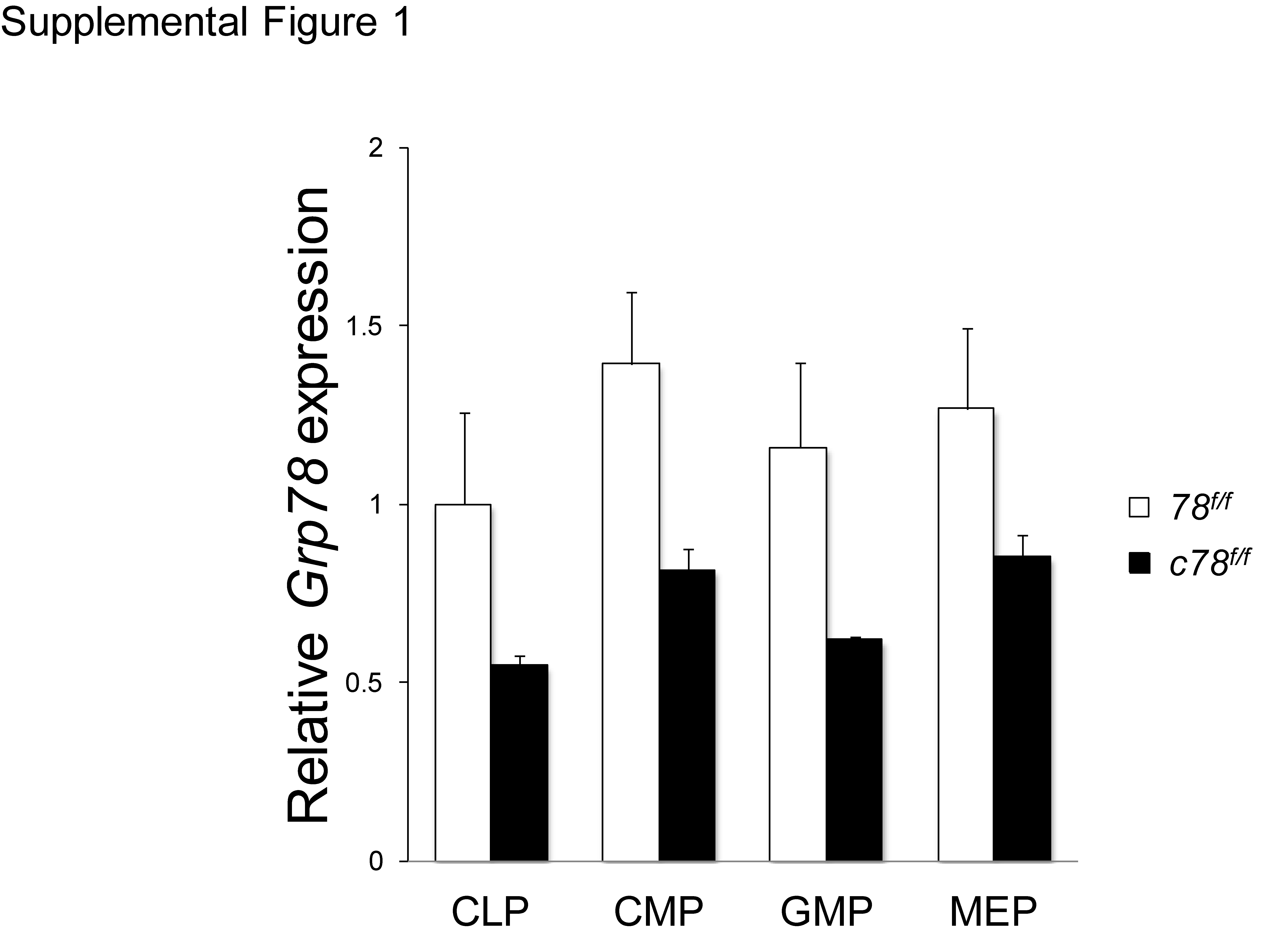

Supplement: Figure S1 — GRP78 knockout efficiency in lymphoid and myeloid progenitors. Bar graph represents the medium intensities of total GRP78 staining measured by flow cytometry in common lymphoid progenitor (CLP), common myeloid progenitor (CMP), granulocyte-monocyte progenitor (GMP) and megakaryocyte-erythroid progenitor (MEP) from 78f/f (n = 2) and c78f/f (n = 2) mice. (TIF) [file pone.0039047.s001.tif]

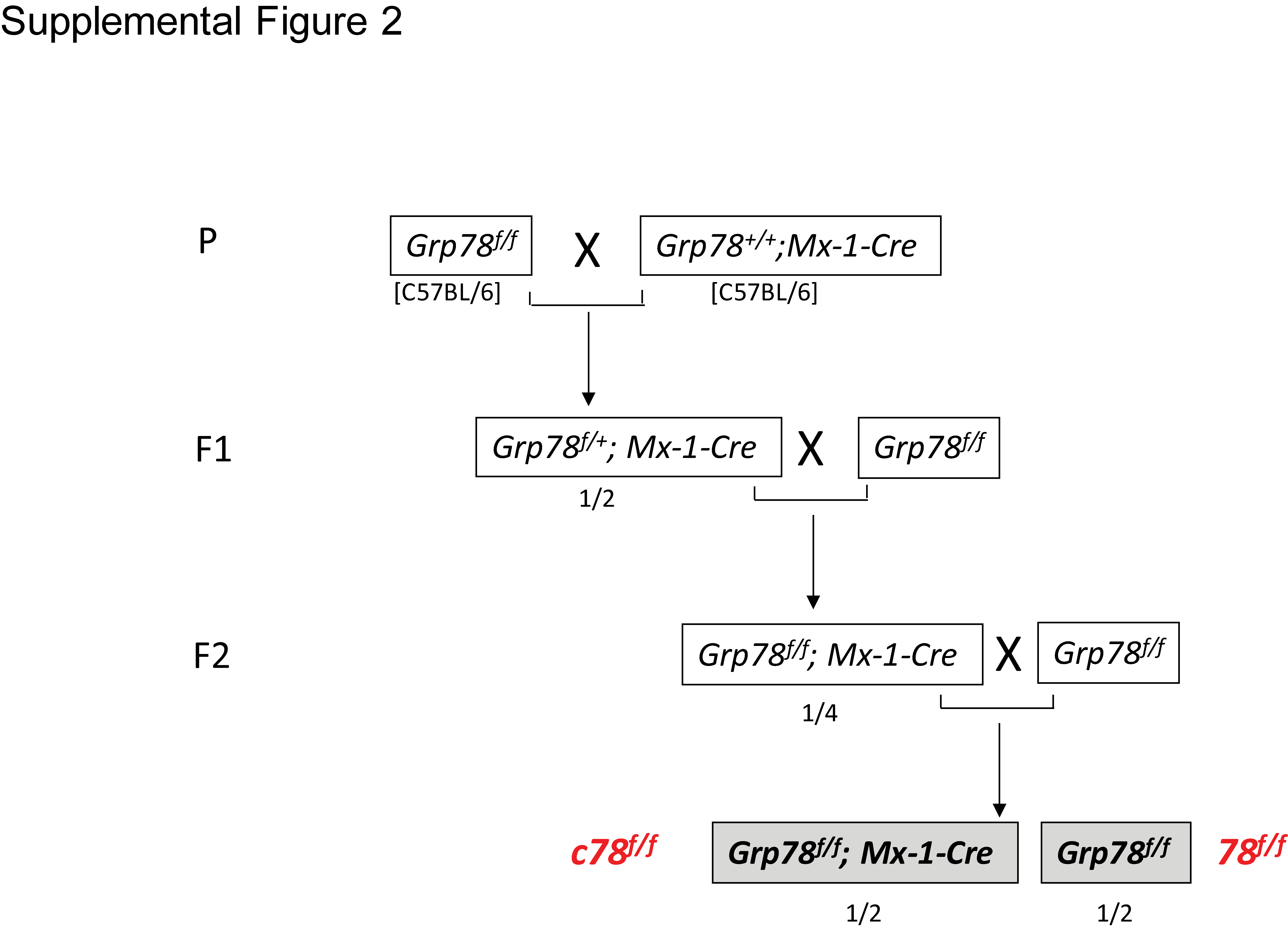

Supplement: Figure S2 — The breeding scheme for the Grp78 conditional knockout mice. The generation of parental Grp78f/f was described previously [11], [14]. Grp78+/+;Mx-1-Cre was commercially purchased from the Jackson Laboratory. The genotypes indicated with the gray shade were used in this study. The genetic background of the parental mouse strain is indicated below within the square brackets. The numbers below the genotypes indicate the expected probability of the indicated genotype among the offspring. (TIF) [file pone.0039047.s002.tif]
