# Supplementary material for: Acute Inducible Ablation of GRP78 Reveals Its Role in Hematopoietic Stem Cell Survival, Lymphogenesis and Regulation of Stress Signaling
Source: PLoS One. 2012 Jun 18;7(6):e39047. doi: 10.1371/journal.pone.0039047 (PMC3377598; doi:10.1371/journal.pone.0039047)
Supplement: File S1 — Materials and Methods. (DOC) [file pone.0039047.s003.doc]

**Supporting Information S1. Materials and Methods**

**Multiplex cytokine assay of serum**

Briefly, a 96-well Multiscreen Resist Vacuum Manifold filter plate (Millipore) was pre-wetted with Bio-plex assay buffer, and multiplex beads were added to the wells. Multiplex beads were washed twice with Bio-plex wash buffer and 50 μl of reconstituted standards or diluted serum samples were added to the wells. Serum samples were diluted 1:3 with mouse serum diluents (Bio-Rad Laboratories). The filter plate was incubated with gentle shaking at 300 rpm for 30 mins at room temperature and washed three times with Bio-plex wash buffer. Bio-plex detection antibody was then added to the wells and incubated with shaking at 300 rpm for 30 mins at room temperature. The filter plate was washed three times with Bio-plex wash buffer and 50 μl Streptavidin-PE was added to each well and incubated with shaking at 10 mins in the dark. The filter plate was washed three times with Bio-plex wash buffer and resuspended in 125 μl of Bio-plex assay buffer and the beads detected using the Bio-plex HTF system (Bio-Rad Laboratories) and results analyzed using the Bio-plex Manager software (Bio-Rad Laboratories).
